# Complete Genome Sequences of Pseudomonas lundensis Strains M101 and M105, Isolated from 1% Pasteurized Milk

**DOI:** 10.1128/MRA.00711-21

**Published:** 2021-10-21

**Authors:** Keerthikka Ravi, John R. Erb-Downward, Natalie K. Gammon, Nicole R. Falkowski, Gary B. Huffnagle

**Affiliations:** a Department of Molecular, Cellular, and Developmental Biology, University of Michigan, Ann Arbor, Michigan, USA; b Division of Pulmonary and Critical Care Medicine, University of Michigan Medical School, Ann Arbor, Michigan, USA; c Department of Microbiology and Immunology, University of Michigan, Ann Arbor, Michigan, USA; d Mary H. Weiser Food Allergy Center, University of Michigan, Ann Arbor, Michigan, USA; University of Arizona

## Abstract

Here, we report the complete genome sequences of two strains of Pseudomonas lundensis, M101 and M105, which were isolated from 1% pasteurized milk. Long-read sequencing was performed using a MinION sequencer, and reads were assembled into circular chromosomes of 4,842,187 bp and 4,814,486 bp for M101 and M105, respectively. Both strains had additional plasmid sequences.

## ANNOUNCEMENT

Pseudomonas lundensis is a Gram-negative, ubiquitous psychrotrophic bacterium that belongs to the Pseudomonas fluorescens complex of species (1, 2). It can thrive in cold environments, such as Antarctic melt ponds, and it is also a well-known cause of cold food spoilage, including milk and dairy products (3). Interestingly, the bacterium has also been isolated from sputum samples from patients with cystic fibrosis (2). To date, very little is known about any source-specific genomic features of P. lundensis. Here, two strains of P. lundensis (M101 and M105) were isolated from 1% pasteurized milk (purchased at a U.S. supermarket) by selective outgrowth for 3 weeks at 4°C. A 16S rRNA gene-specific colony PCR assay, a P. lundensis-specific (P. lundensis ExoU) PCR assay, and average nucleotide identity analysis with BLAST were used to confirm the identities of the strains as P. lundensis (4). Here, we present the complete genome sequences of these two milk isolates of P. lundensis.

Frozen (−80°C) glycerol stocks of the isolated strains were grown in LB medium at room temperature, and DNA isolation for whole-genome sequencing was performed using the DNeasy blood and tissue kit (Qiagen) without RNase treatment.

Sequencing libraries were prepared using the rapid barcoding kit (SQK-RBK001) and sequenced on an R9.4.1 flow cell for 4 h using a MinION sequencer (Oxford Nanopore Technologies). Base calling for the raw reads was performed using Guppy v4.2.3 in high-accuracy mode. A total of 79,480 and 58,783 reads, with *N*_50_ values of 5,855 and 6,460 bp, respectively, were obtained after base calling for M101 and M105, respectively. The genome sequences were assembled *de novo* using Flye v2.8.1 (5) with default parameters. The assembly was further polished through two rounds with Racon v1.4.20 (6) using default parameters for all except scores for matching and mismatching bases (-match 8 -mismatch -6). A final round of polishing was performed using Medaka v1.2.3 (7), once again using default parameters.

M101 (genome coverage, 53×) and M105 (genome coverage, 44×) were assembled into three and two circular contigs, respectively (Table 1). The assembled contigs were confirmed as nonviral contigs using ViralVerify (8). plasmidVerify (9) identified the 4.8-Mb contigs in both M101 and M105 as chromosomes and the two 24-kb contigs, as well as the 3-kb contig, as plasmid sequences. Sequence annotation for both strains was performed using the NCBI Prokaryotic Genome Annotation Pipeline (PGAP) (10).

**TABLE 1:** Genome sequence data for Pseudomonas lundensis isolates M101 and M105

Assembly accession no.	Strain	SRA accession no.	GenBank accession no.	Contig	Genome size	GC content (%)
GCA_018449005.1	M101	SRR15403967	CP075177.1	Chromosome	4.8 Mb	58.69
			CP075178.1	pPL-3mi	3 kb	60.4
			CP075179.1	pPL-24mi	24 kb	55.4
GCA_018449025.1	M105	SRR15403966	CP075180.1	Chromosome	4.8 Mb	58.69
			CP075181.1	pPL-24mi	24 kb	55.4

### Data availability.

The raw sequence data and assembled sequences are deposited in NCBI under the BioProject accession number PRJNA729724. The accession numbers for the deposited sequences are listed in Table 1.
